# Bioinformatics Analysis Identifies EPAS1 as a Novel Prognostic Marker Correlated with Immune Infiltration in Acute Myeloid Leukemia

**DOI:** 10.1155/2023/6072782

**Published:** 2023-04-17

**Authors:** Shichun Wang, Pengyu Zhang

**Affiliations:** Department of Blood Transfusion, Tianjin Medical University Cancer Institute & Hospital, National Clinical Research Center for Cancer, Tianjin's Clinical Research Center for Cancer, Tianjin, China

## Abstract

EPAS1 plays an important role in the development and progression of multiple tumor types by interacting with a series of other molecules. However, the prognostic and diagnostic values of EPAS1 in acute myeloid leukemia (AML) remain unknown. Here, we systematically explored and clarified the potential functions of EPAS1 in AML using data from Xena Browser and TCGA database. The expression of EPAS1 was significantly lower in AML patients than that in healthy people. The GO, KEGG, GSEA, and GSVA were performed to explore the potential functions and signaling pathways. The survival analysis was conducted using Cox regression analysis and the Kaplan-Meier method. Immune cell infiltration was evaluated via single-sample GSEA (ssGSEA). The results of enrichment analyses suggested that low-EPAS1 expression was related to the initiation, development, and prognosis of AML. The immune microenvironment landscape in AML was described by ssGSEA. ROC analysis of EPAS1 showed high discrimination ability between AML patients and healthy people. Kaplan-Meier method indicated that low-EPAS1 expression correlated significantly with a poor overall survival. Multivariate Cox regression analysis revealed that both age and EPAS1 expression were independent prognostic factors in AML patients. Furthermore, the nomogram based on these two variables performed well in discrimination and calibration. In summary, our study may provide new insights into the molecular mechanisms underlying AML and demonstrate the diagnostic and prognostic value of EPAS1 in AML for the first time.

## 1. Introduction

Acute myeloid leukemia (AML) is an aggressive hematologic neoplasm characterized by abnormal proliferation and differentiation of hematopoietic precursors, resulting in the accumulation of leukemic cells in the bone marrow and peripheral blood. There are estimated 19940 new AML cases and 11180 deaths in 2020, making it the most common and deadliest form of acute leukemia in adults [1]. The 5-year relative survival for AML patients declines with increasing age, from 47.5% in patients younger than 65 years old to just 8.2% in those aged 65 years and older [2]. With the continuous advancement in conditional treatments for AML, including chemotherapy, allogeneic stem cell transplantation (SCT), and supportive care, the outcome improvement in younger patients has been reported in several trials [3]. While in elderly patients who cannot tolerate intensive chemotherapy regimens, the effect is not satisfactory [3–5]. Thus, it is an urgent need to explore potential prognostic markers and therapeutic targets that can contribute to patient-tailored treatment plan making and clinical management.

EPAS1, the gene encoding hypoxia-inducible factor- (HIF-) 2*α*, plays a critical role in a wide range of pathophysiological processes. Genome-wide studies have shown that the mutation of EPAS1 is closely linked to human adaptation to high-altitude hypoxia, with a particular focus on Tibetans [6]. There is growing evidence showing that EPAS1 is related with cancer initiation and progression. Mutations in EPAS1 are considered to be drivers in pheochromocytoma and paraganglioma [7–9]. In renal cell carcinoma (RCC), the HIF-2*α* regulation is linked to the cancer development in vivo and in vitro [10–12]. And preclinical studies and clinical data validate that HIF-2*α* inhibitors have antitumor activity in RCC cell lines and heavily pretreated patients [13–16]. On the other hand, HIF-2*α* has been reported to suppress tumor growth in undifferentiated pleomorphic sarcoma, fibrosarcoma, and dedifferentiated liposarcoma, where EPAS1 is largely considered to be epigenetically silenced [17]. In addition, HIF-2*α* is found to contribute to the tumor cell apoptosis in hepatocellular carcinoma through the TFDP3/E2F1 pathway, and lower HIF-2*α* expression level in tissue is always accompanied by poorer survival [18]. These findings suggest the potential role of EPAS1 as a therapeutic target and as a prognostic marker for patients with cancers. However, the underlying functions and molecular mechanisms of EPAS1 in AML are still poorly understood. In this research, we aimed to determine whether EPAS1 could serve as a prognostic marker to guide clinical decision-making in AML and explore the role of EPAS1 in AML disease mechanism, which is the key to identify novel diagnostic and therapeutic avenues.

## 2. Materials and Methods

### 2.1. Study Design

**Figure 1:**
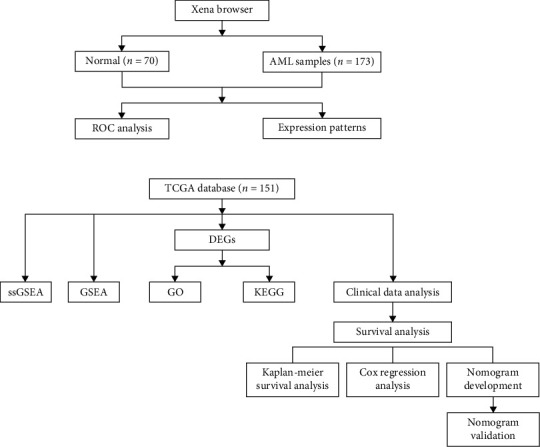
Overall flow chart of this work.

Gene expression data and clinical information were extracted from the common databases. Then, the computational biology tools were applied to evaluate the diagnostic and prognostic values of EPAS1 in AML patients. The R code used for our analysis is available in Supplementary Materials. The schematic flow chart of the present study was summarized in Figure 1.

### 2.2. Preprocessing for RNA-Sequencing Data

Genotype-Tissue Expression (GTEx) TOIL RSEM transcript per million (tpm) data, TCGA Pan-Cancer TOIL RSEM tpm data, and corresponding clinical information were obtained from Xena Browser (https://xenabrowser.net/datapages/) [19]. The data of 70 GTEx normal samples and 173 TCGA-AML samples were processed through TOIL in a uniform manner and then transformed into log_2_(TPM + 1) for differential expression analysis and receiver operating characteristic (ROC) analysis.

The clinical data of 200 AML patients were obtained from TCGA database (https://portal.gdc.cancer.gov/repository). Cases without corresponding RNA-seq data (*n* = 49) were excluded. Finally, we compiled log_2_(TPM + 1) gene expression data of 151 AML cases for further analysis. The data were collated into Table 1, and unavailable or unknown clinicopathological features were treated as missing values. This study is entirely based upon data generated from TCGA and meets the TCGA publication guidelines. (http://www.cancer.gov/about-nci/organization/ccg/research/structural-genomics/tcga). This article does not contain any studies with human participants or animals performed by any of the authors.

### 2.3. Differentially Expressed Gene (DEG) Analysis

According to the median expression level of EPAS1, gene expression data were divided into a high-expression group and a low-expression group. To identify the DEGs in AML, we analyzed and compared the expression data between high- and low-EPAS1 expression groups within the DESeq2 package (version 1.28.1) [20]. Genes with |log_2_ fold change| > 1.5 and adjusted *P* value < 0.05 were considered to be statistically significant. The results were visualized using volcano plots and heat map.

### 2.4. Functional Enrichment Analysis

Metascape (http://metascape.org) is a network-based tool integrating gene annotation, enrichment analysis, and interactome analysis capabilities [21]. In this study, we used Metascape to conduct Gene Ontology (GO) and Kyoto Encyclopedia of Genes and Genomes (KEGG) enrichment analysis for EPAS1-related DEGs in AML. Similar terms were grouped into a cluster under the parameters that *P* value < 0.01, minimum count > 3, and enrichment factor > 1.5.

### 2.5. Gene Set Enrichment Analysis

Gene Set Enrichment Analysis (GSEA) is a powerful and robust analytical strategy designed to reveal the collective behavior of functionally related genes by identifying biological pathways at the gene-set level, which makes it easier for researchers to interpret the results in large-scale analysis [22, 23]. GSEA takes all gene expression data as analysis objects to avoid the loss of key information caused by unreasonable filtering parameters in traditional enrichment analysis. In this study, GSEA was carried out using clusterProfiler (version 3.16.0) package in R (3.8.0) [24] to elucidate the significant differences in function or pathway between high- and low-EPAS1 expression groups. Gene set permutations were performed 1000 times for each analysis. The enrichment analysis was based on the conditions that adjusted *P* value < 0.05, FDR *q* value < 0.25, and |NES| > 1.

### 2.6. Gene Set Variation Analysis

Gene set variation analysis (GSVA) [25] is a nonparametric analysis method to evaluate whether different metabolic pathways are enriched in different samples by converting the gene expression matrix between different samples into gene set expression matrix. The gene sets (c2.cp.v2022.1.Hs.symbols) were obtained from the MSigDB database. The 151 samples from the TCGA-AML dataset were divided into high-expression group and low-expression group according to the median expression level of EPAS1. Enrichment scores were calculated for each gene set to evaluate the potential biological function changes of different samples using the GSVA algorithm. The criterion of significant enrichment was adjusted *P* value < 0.05.

### 2.7. Immune Infiltration Analysis by ssGSEA

Using GSVA package (http://www.bioconductor.org/packages/release/bioc/html/GSVA.html) in R, the immune infiltration analysis was performed by single-sample GSEA (ssGSEA). We quantified the relative enrichment scores of 24 tumor-infiltrating immunocytes in AML by comparing the published signatures [26] with the gene expression profile data. The correlation between EPAS1 expression and infiltration levels of these immunocytes and the association of the high- and low-EPAS1 expression groups with the infiltration of immune cells were analyzed by Spearman correlation and Wilcoxon rank sum test, respectively.

### 2.8. Statistical Analysis

All statistical analyses in our study were conducted using R (v.3.6.3). We analyzed the different expressions of EPAS1 in AML and healthy control groups with the Wilcoxon rank sum test and assessed the discrimination ability of EPAS1 by receiver operating characteristic (ROC) analysis using pROC package [27]. Wilcoxon rank sum test and logistic regression were applied to analyze the correlation between clinicopathologic features and EPAS1 expression. The survival analysis was conducted using Cox regression analysis and the Kaplan-Meier method with 95% confidence intervals (CI) [28]. The hazard ratios (HRs) of overall survival (OS) in subgroups were summarized in forest plot. All hypothetical tests were two-sided, and *P* values less than 0.05 were considered to be statistically significant.

### 2.9. Prognostic Model

We applied multivariate Cox regression analysis to screen out independent variables related to survival. Furthermore, a nomogram and calibration plots were constructed using rms package (version: 6.2-0, https://cran.r-project.org/web/packages/rms/index.html) to predict the prognosis of AML patients.

## 3. Results

### 3.1. Patient Characteristics

The characteristics of AML patients with both gene expression and clinical data obtained from TCGA database were shown in Table 1. Correlation analysis showed that the expression level of EPAS1 was significantly associated with cytogenetic risk (*P* = 0.001), FAB classification (*P* = 0.048), cytogenetics (*P* = 0.002), FLT3 mutation (*P* = 0.026), NPM1 mutation (*P* = 0.001), WBC count (*P* = 0.001), BM blasts (*P* < 0.001), and PB blasts (*P* = 0.001). The distributions of other clinicopathologic features were not different between low- and high-EPAS1 expression groups.

### 3.2. Identification of DEGs

**Figure 2:**
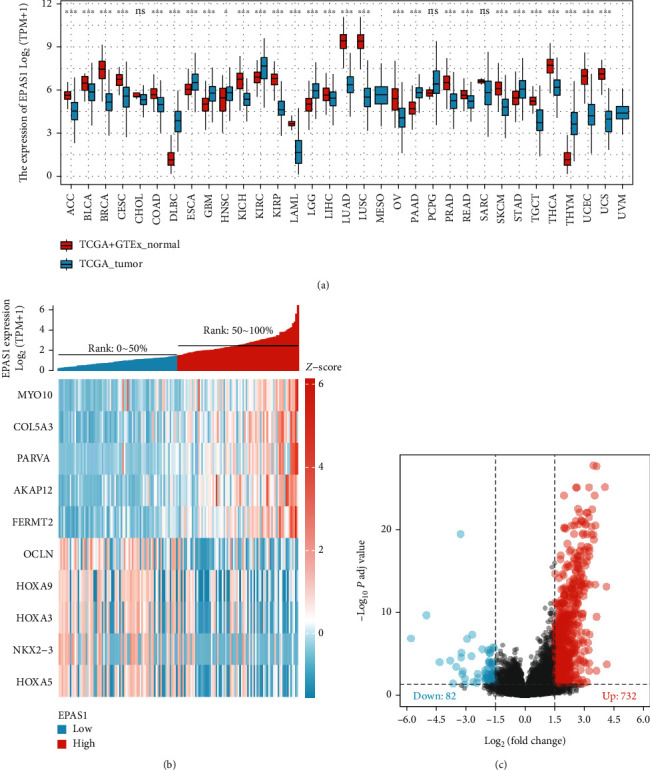
Identification of differentially expressed genes between low- and high-EPAS1 expression groups in TCGA database. (a) EPAS1 expression in different types of cancer. (b) Heat map of differentially expressed genes between low- and high-EPAS1 expression groups. (c) Volcano plot of differentially expressed genes. Red dots represent the upregulated genes, and blue dots represent the downregulated genes. The gray area represents the genes whose expression levels are below the threshold (|log_2_fold change| > 1.5, adjusted *P* value < 0.05).

Under the aforementioned threshold (|log_2_ fold change| > 1.5, adjusted *P* value < 0.05), 814 DEGs, including 732 upregulated DEGs and 82 downregulated DEGs, were identified between low- and high-EPAS1 expression groups (Figure 2).

### 3.3. GO and KEGG Analyses of EPAS1 in AML

**Figure 3:**
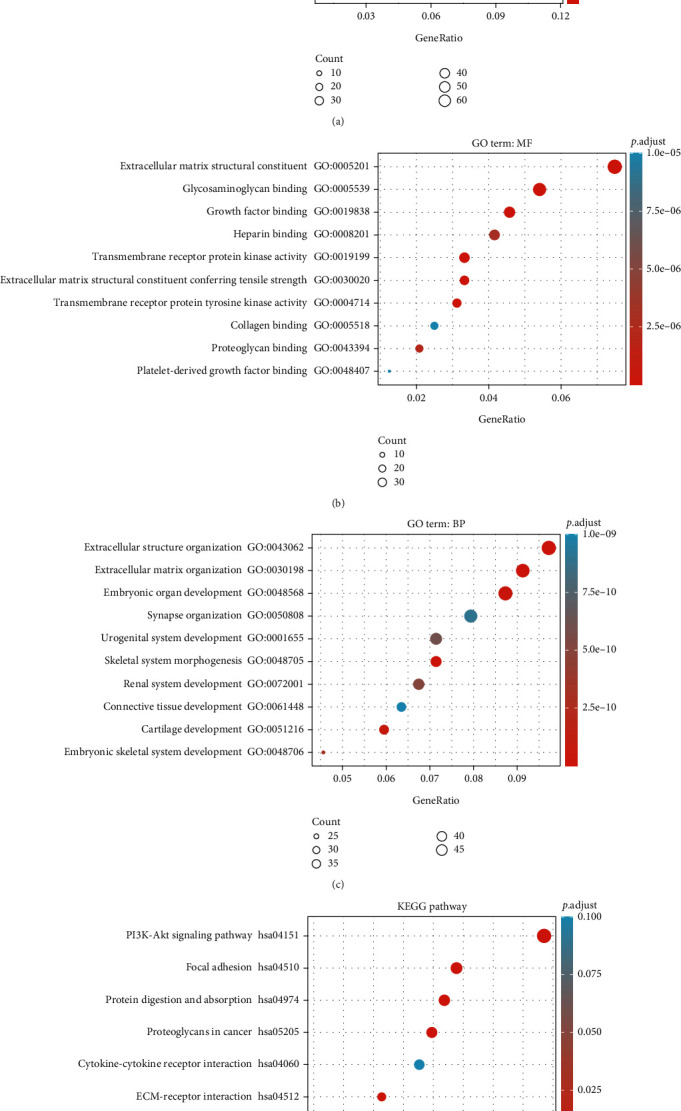
GO and KEGG enrichment analyses for EPAS1 (Metascape). (a–c) Top 10 enrichment terms in cellular component (CC), molecular function (MF), and biological process (BP) categories in AML. (d) Bubble plot of KEGG enrichment pathways colored by adjusted *P* values.

Enrichment analyses were conducted within Metascape to obtain the enrichment information of EPAS1-related DEGs in AML. The top 10 GO terms enriched by DEGs are shown in Figures 3(a)–3(c). The results of cellular components (CC) suggested that EPAS1-related genes were mainly located in extracellular matrix. The molecular functions (MF) of these genes included extracellular matrix structural composition, molecular binding, and protein kinase activation. Moreover, we found that these genes were involved in several biological processes (BP), including extracellular structure organization, extracellular matrix organization, system morphogenesis, and tissue or organ development. The KEGG pathways for EPAS1 and its correlated genes are shown in Figure 3(d).

### 3.4. EPAS1-Related Signaling Pathways Based on GSEA

**Figure 4:**
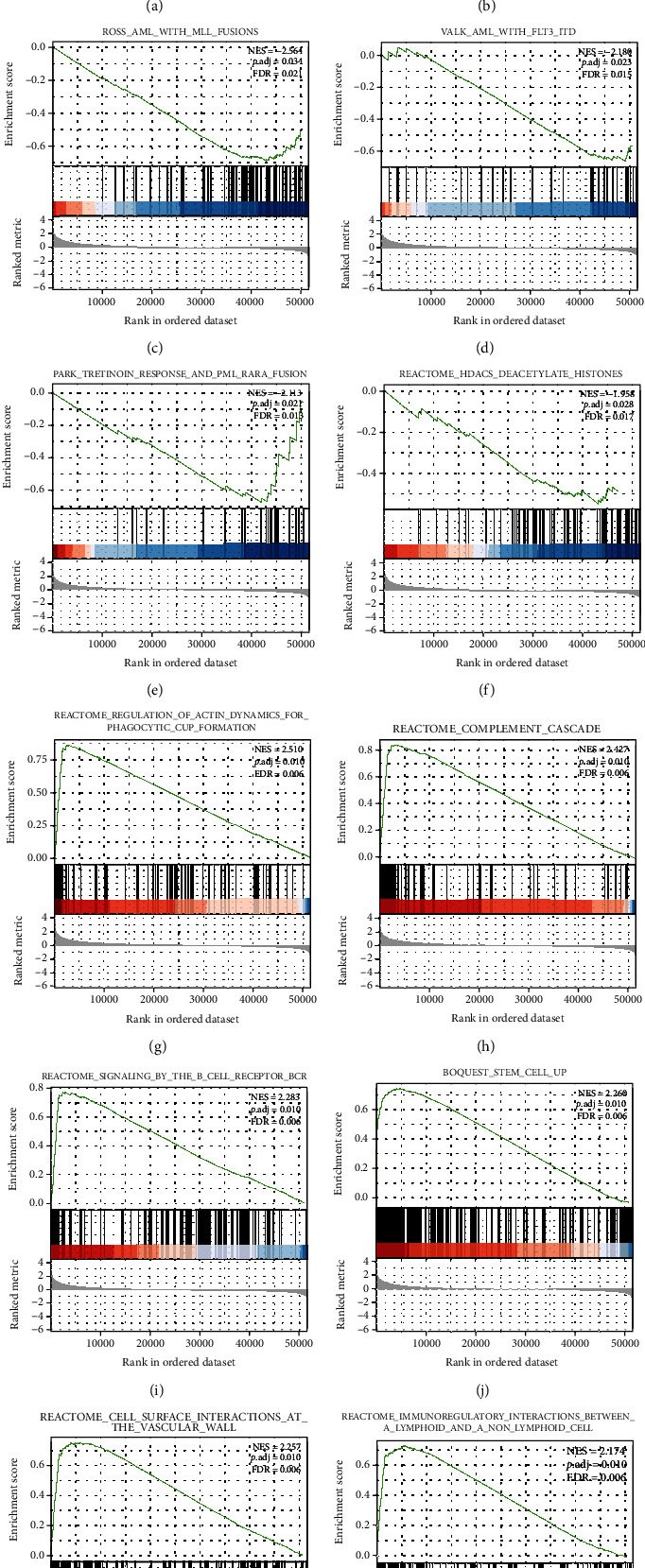
Enrichment plots from Gene Set Enrichment Analysis (GSEA). Ribosome, SRP-dependent cotranslational protein targeting to membrane, AML with MLL fusions, AML with FLT3-ITD, tretinoin response and PML-RARA fusion, and HDACS deacetylate histones are differentially enriched in low-EPAS1 expression phenotype. Regulation of actin dynamics for phagocytic cup formation, complement cascade, signaling by the B cell receptor, stem cell up, cell surface interactions at the vascular wall, and immunoregulatory interactions between a lymphoid and a nonlymphoid cell are differentially enriched in high-EPAS1 expression phenotype. SRP: signal recognition particle; ITD: internal tandem duplication; HDACS: histone deacetylase; NES: normalized enrichment score; FDR: false discovery rate.

To identify the key signaling pathways stimulated differentially in AML, we performed GSEA between low- and high-EPAS1 expression data sets. Significant differences (adjusted *P* value < 0.05, FDR *q* value < 0.25) in the enrichment of MSigDB collection (C2.all.v7.0.symbols.gmt) were revealed. Based on their NES, we selected biological processes and pathways that were significantly enriched in the low-EPAS1 expression phenotype, including ribosome, SRP-dependent cotranslational protein targeting to membrane, AML with MLL fusions, AML with FLT3-ITD, tretinoin response and PML-RARA fusion, and HDACS deacetylate histones. The details are shown in Figures 4(a)–4(f) and Table 2. We also selected biological processes and pathways that were significantly enriched in the high-EPAS1 expression phenotype, including regulation of actin dynamics for phagocytic cup formation, complement cascade, signaling by the B cell receptor, stem cell up, cell surface interactions at the vascular wall, and immunoregulatory interactions between a lymphoid and a nonlymphoid cell. The details are shown in Figures 4(g)–4(l) and Table 3.

### 3.5. GSVA Analysis of EPAS1 in AML

In order to explore the differences of curated gene sets between high- and low-EPAS1 expression groups in TCGA-AML dataset, the analyses were performed by ssGSEA using GSVA package. According to the enrichment scores, the differences in extracellular matrix-related pathways between these two groups were shown by the boxplot in Supplementary Figure 1. In the high EPAS1 expression group, the enrichment scores of extracellular matrix-related pathways, including degradation of the extracellular matrix, extracellular matrix organization, cell extracellular matrix interactions, extracellular vesicle-mediated signaling in recipient cells, and extracellular vesicles in the crosstalk of cardiac cells, were significantly higher than that in the low-EPAS1 expression group (*P* value < 0.05). We further showed the top 25 genes significantly differentially expressed in high-EPAS1 expression group, low-EPAS1 expression group, and normal control group by heat map in Supplementary Figure 2 (*P* value < 0.05). Heat map was generated using R pheatmap package.

### 3.6. The Correlation between EPAS1 Expression and Immune Infiltration

**Figure 5:**
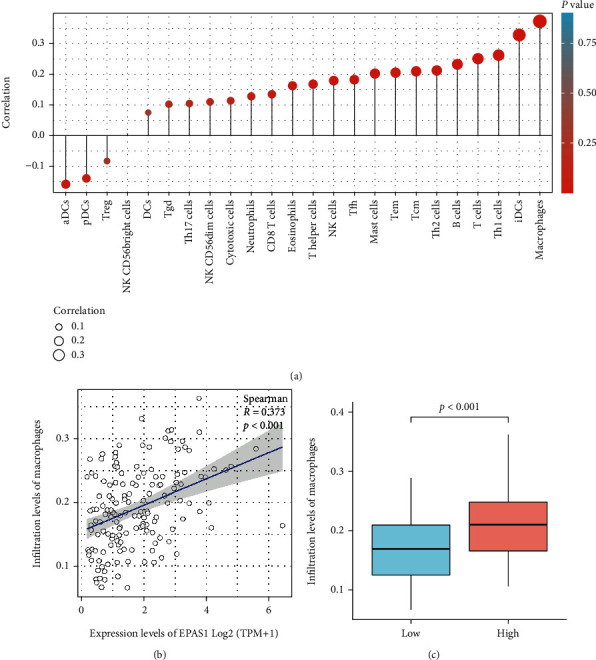
EPAS1 expression level was associated with immune filtration in AML. (a) Correlations of EPAS1 expression with abundances of 24 immune cells. The dots are colored by *P* value, and their size represents the value of Spearman r. (b, c) Correlation between the expression level of EPAS1 and the infiltration level of macrophages.

Spearman correlation test was applied to assess the correlation between EPAS1 expression level (TPM) and immune cell infiltration levels which were quantified by comparing with immunocyte signatures using ssGSEA. The EPAS1 expression was positively correlated with macrophages and immature dendritic cells (iDCs) (Figure 5(a)). The correlation between macrophages and EPAS1 expression was strongest among the 24 tumor-infiltrating immunocytes (Spearman *r* = 0.373, *P* value < 0.001) (Figure 5(b)). Wilcoxon rank sum test showed that the abundance of macrophages in low-EPAS1 expression group was significantly lower than that in high-EPAS1 expression group with *P* value < 0.001 (Figure 5(c)).

### 3.7. Expression Patterns of EPAS1 in AML

**Figure 6:**
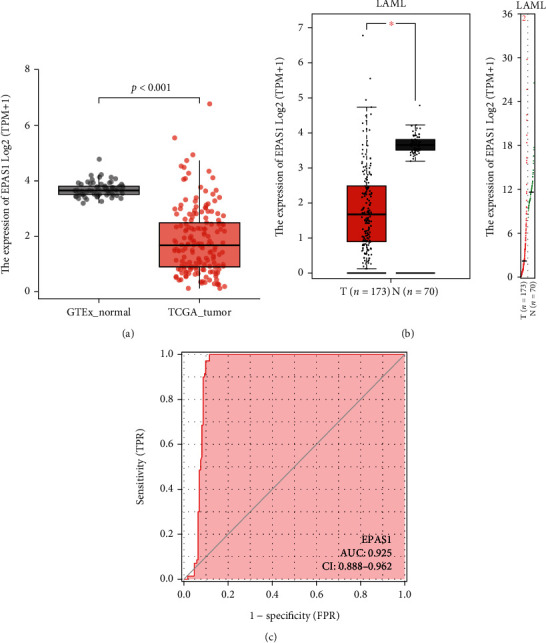
Expression patterns of EPAS1 in AML. (a) Lower EPAS1 expression in AML group than that in control group (*P* < 0.001). (b) As shown in the box plot (up) and scatter diagram (down), the expression level of EPAS1 is lower in AML than that in control (Gene Expression Profiling Interactive Analysis (GEPIA)). (c) ROC analysis of EPAS1 showing a high discrimination ability between AML patients and healthy people (AUC = 0.925). ROC: receiver operating characteristic; AUC: area under curve.

Wilcoxon rank sum test showed that there was a significant difference in EPAS1 expression between AML patients and healthy person. The expression level of EPAS1 was significantly lower in AML group than that in control group (*P* < 0.001; Figures 6(a) and 6(b)). We then evaluated the discrimination ability of EPAS1 between two groups by receiver operating characteristic (ROC) analysis. The AUC = 0.925 indicated that EPAS1 had a high discrimination value for AML (Figure 6(c)).

**Figure 7:**
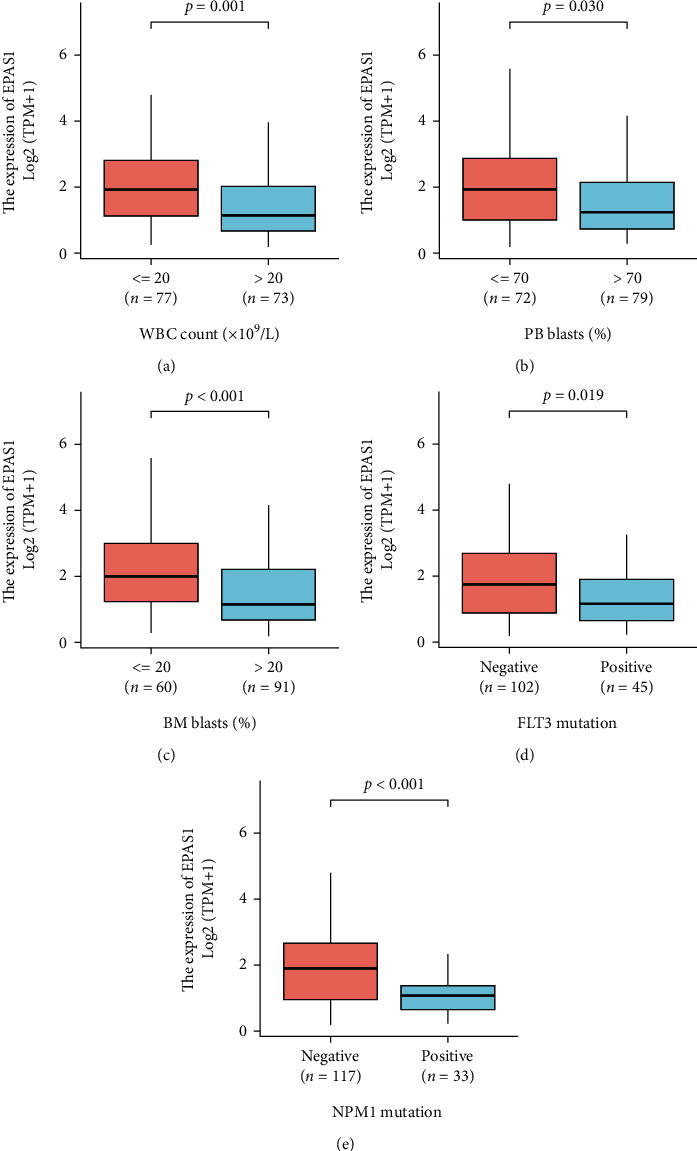
(a–e) Association of EPAS1 expression and clinicopathologic variables including (a) WBC count, (b) PB blasts, (c) BM blasts, (d) FLT3 mutation, and (e) NPM1 mutation. PB: peripheral blood; BM: bone marrow.

Univariate logistic regression analysis revealed that EPAS1 expression based on median value was associated with clinicopathologic variables. As shown in Figures 7(a)–7(e) and Table 4, decreased expression of EPAS1 in AML was significantly associated with WBC count (×10^9^/L) (OR = 0.42 for >20 vs. <=20, *P* = 0.009), PB blasts (%) (OR = 0.46 for >70 vs. <=70, *P* = 0.019), BM blasts (%) (OR = 0.31 for >20 vs. <=20, *P* < 0.001), FLT3 mutation (OR = 0.41 for positive vs. negative, *P* = 0.017), and NPM1 mutation (OR = 0.20 for positive vs. negative, *P* < 0.001).

### 3.8. Role of EPAS1 in AML Patient Survival

**Figure 8:**
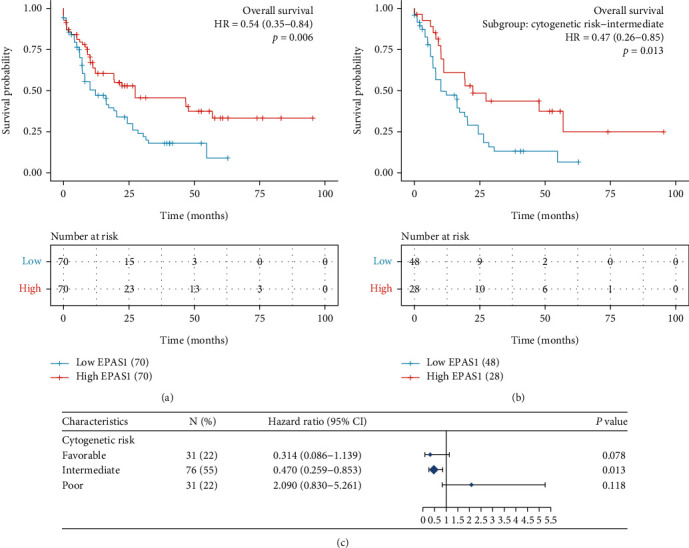
(a) Low expression of EPAS1 is associated with poor over survival (OS) in patients with AML (*P* = 0.006). (b) Low expression of EPAS1 is associated with poor OS in the subgroup of intermediate cytogenetic risk (*P* = 0.013). (c) Forest plot of OS hazard ratios for key subgroups.

Kaplan-Meier survival analysis revealed that OS was significantly poorer in patients with low-EPAS1 expression than those with high-EPAS1 expression (*P* = 0.006) (Figure 8(a)). In OS subgroup analysis, we found that low expression of EPAS1 was associated with poor OS in the subgroup of intermediate cytogenetic risk (*P* = 0.013) (Figures 8(b) and 8(c)). The univariate Cox regression analysis showed that low-EPAS1 expression correlated significantly with a poor OS (HR: 0.542; 95% CI: 0.352-0.836; *P* = 0.006). Other clinicopathologic features, including cytogenetic risk and age, were associated with poor outcome as well. At multivariate analyses, low-EPAS1 expression was still independently correlated with OS (HR: 0.577; 95% CI: 0.364-0.915; *P* = 0.019), along with age (HR: 3.174; 95% CI: 2.023-4.980; *P* < 0.001) (Table 5).

### 3.9. Development and Validation of Nomogram

**Figure 9:**
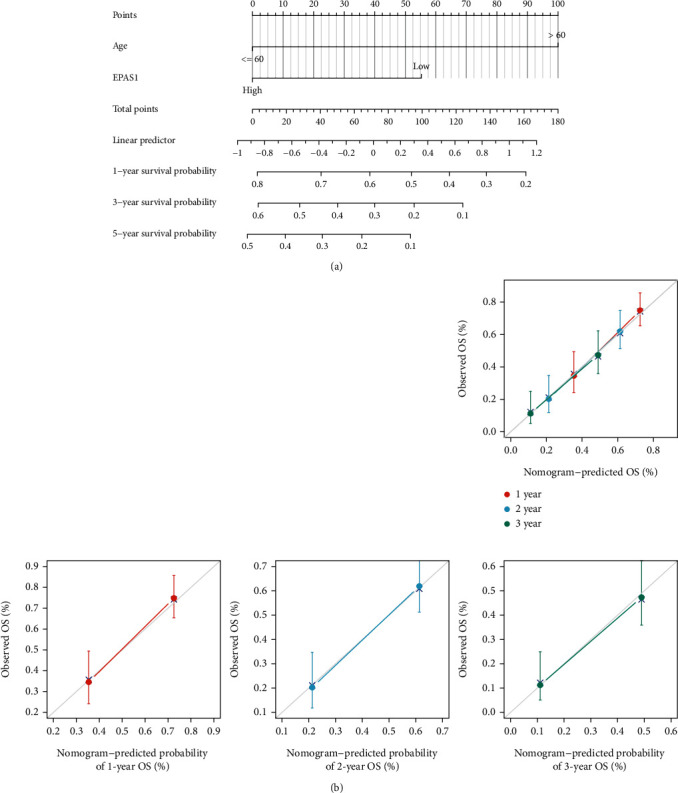
Construction and validation of the nomogram for AML patients. (a) Nomogram for predicting the probability of 1-, 3-, and 5-year over survival (OS) for AML patients. (b) Calibration plot of the nomogram for predicting the probability at 1, 2, and 3 years.

Low-EPAS1 expression and age were determined to be independent prognostic factors for AML by univariate and multivariate Cox regression analyses. Then, we constructed a nomogram-integrated EPAS1 and age. Higher total points represented worse survival probability at 1, 3, and 5 years (Figure 9(a)). The C-index for the nomogram was 0.714 (95% CI: 0.689-0.739). Lines in calibration plot were all close to the ideal line, indicating that the predictions made by nomogram conformed well to the observations in AML patients (Figure 9(b)).

## 4. Discussion

Hypoxia-inducible factors (HIFs) mediate the responses to oxygen (O_2_) concentrations in cells, which makes the regulatory role of HIFs in tumor development and progression become an intensive area of research. EPAS1 (HIF-2), a heterodimeric member of the basic helix-loop-helix-PAS domain polypeptide family, consists of *α* and *β* subunits [29]. EPAS1 shares 48% identity with HIF-1*α* whose regulatory functions in glucose metabolism, cell proliferation, angiogenesis, tumor invasion, and survival have been elucidated over the past decades [30]. Although it is tempting to speculate that EPAS1 is functionally similar to HIF-1*α*, accumulating evidence shows that EPAS1 and HIF-1*α* could lead to nonequivalent and even opposite effects on solid tumor development, progression, and prognosis [31]. For example, in von Hippel-Lindau-defective renal cell carcinoma (RCC), HIF-1*α* inhibits cell proliferation in vivo or in vitro, and the knockdown of HIF-1*α* promotes tumor growth accordingly [32], while EPAS1 is shown to be essential for the growth of xenograft in RCC [11]. In hematological malignancies, HIF-1*α* was required for the maintenance of cancer stem cells in AML, and the downregulation of HIF-1*α* could effectively eliminate the activity of AML colony-forming unit [33]. However, few studies have addressed the role of EPAS1 in AML.

The complex interplay of genetic events has long been considered as the basis for disease classification, risk stratification, and prognostic assessment in AML [34, 35]. This is basically consistent with the results of our EPAS1-related GSEA analysis, including biological processes such as leukemia-associated genetic alterations, epigenetic regulation, and immune microenvironment changes. Noteworthy, both GSEA and logistic regression analysis showed that the low-level EPAS1 expression was significantly correlated with FMS-like tyrosine kinase-3 (FLT-3) mutation, which is considered to be one of the most important drivers in AML [36], being associated with poor prognosis in AML [37]. In the absence of ligands, FLT-3 mutations constitutively activate of FLT-3 receptors, promoting cell proliferation, and survival through PI3K/AKT and other signaling pathways. Interestingly, the results of GO and KEGG analyses pointed in a similar direction. A number of studies have shown that EPAS1 is closely related to the PI3K/AKT signaling pathway. Researchers have elucidated the complex regulatory relationship between EPAS1 and PI3K/AKT signaling pathway in different cancers, in which factors such as PTEN, YY1, SCD1, and CD44 also play important roles [38–40]. Although little is known about the regulatory mechanism between EPAS1 and PI3K/AKT signaling pathway in hematological malignancies, we can gain information and inspiration from these similar studies in solid tumors. In addition, the results of ROC curve analysis in our study also suggested that EPAS1 had a good discriminative ability for AML. Taken together, all the findings indicate that further studies on EPAS1 may contribute to personalized treatment and development of targeted drugs for patients with AML.

Age has been previously reported as an independent prognostic factor for AML [41], which is consistent with our results obtained by multivariate Cox regression analysis. From the statistical data in recent years, it is not difficult to see that the majority of AML patients are elderly, whose outcomes are particularly poor [42, 43]. In view of this, the development of prognostic prediction methods is particularly urgent and important. In our study, the findings from different analytic approaches, including Kaplan-Meier survival analysis, model building, and model validation, corroborated each other and highlighted the potential power for our prognostic model to enhance patient outcome predictions. This suggests that our model may help to determine prognosis and develop treatment plans accordingly in clinical practice and may aid in clinical risk stratification.

AML arises in an immunosuppressive bone marrow microenvironment characterized by promoting tumor growth and immune escape [44]. Interest in the use of immunotherapy to improve AML outcomes is growing due to significant advances in immunotherapy in solid tumors. At present, several immunotherapies are under development and clinical testing [45]. In our study, there is a positive relationship between EPAS1 expression level and infiltration level of many immune cells, particularly macrophages. It is well known that macrophages are derived from monocytes under physiological conditions and regulate the innate and adaptive immune system by secreting cytokines and chemokines. In AML, a large number of immature myeloid cells proliferate, resulting in catastrophic bone marrow failure, which may explain why there was a decrease in macrophage expression. We also observed that the abundance of active dendritic cells (aDCs) in low-EPAS1 expression group was significantly higher than that in high-EPAS1 expression group, although the correlation between aDCs and EPAS1 expression was weak. When DCs are activated, they migrate to lymphoid tissues to interact with T to stimulate and control the appropriate immune response. The increased level of aDCs in low-EPAS1 expression group (AML-patient group) appears to compensate for the tumor immune dysfunction caused by macrophage depletion to some extent. Our results suggest a possible regulatory mechanism of the tumor immune microenvironment associated with low-EPAS1 expression in AML, which may be helpful to the development of immunotherapy for AML patients in the future. However, more well-designed clinical trials are needed to confirm whether and how the infiltration degree of these immune cells changes throughout the course of AML.

There were some limitations in our study. First, all the data were extracted from the public database. The small and unbalanced sample sizes were used in the analyses, so further multicenter and large sample size studies are needed. Second, the current study was performed based on RNA-sequencing data from public database; therefore, we cannot gain deeper insights of the gene function at other omics levels. Finally, it would be better to further collect clinical data on the basis of retrospective analysis for validation. We are collecting bone marrow samples from clinical AML patients for subsequent immunohistochemistry and single-cell sequencing analyses, which will serve as a supplement to our research results and enhance the persuasiveness of our article.

## 5. Conclusions

In conclusion, our study revealed that decreased EPAS1 expression could be a potential diagnostic indicator and a prognostic marker of poor survival in AML. Further experiments are needed to validate our findings and identify the underlying molecular mechanism of AML.

## Figures and Tables

**Table 1:** Demographic and clinicopathological characteristics of AML patients based on EPAS1 expression level.

Characteristics	Frequency (%)	EPAS1 expression		*P*
		Low (*n* = 76)	High (*n* = 75)	
Age		56.00 [43.75,64.25]	57.00 [41.50,69.00]	0.549^b^
Gender				0.284
Female	68 (45.0)	38 (50.0)	30 (40.0)	
Male	83 (55.0)	38 (50.0)	45 (60.0)	
Race				0.564^a^
Asian	1 (0.7)	1 (1.3)	0 (0.0)	
Black or African American	13 (8.7)	8 (10.5)	5 (6.8)	
White	135 (90.6)	67 (88.2)	68 (93.2)	
Cytogenetic risk				0.001^∗^
Favorable	31 (20.8)	8 (10.7)	23 (31.1)	
Intermediate	82 (55.0)	52 (69.3)	30 (40.5)	
Poor	36 (24.2)	15 (20.0)	21 (28.4)	
Cytogenetics				0.002^∗^^,a^
+8	8 (5.9)	3 (4.2)	5 (7.8)	
Complex	24 (17.8)	10 (14.1)	14 (21.9)	
del (5)	1 (0.7)	1 (1.4)	0 (0.0)	
del (7)	6 (4.4)	3 (4.2)	3 (4.7)	
inv (16)	8 (5.9)	0 (0.0)	8 (12.5)	
Normal	69 (51.1)	46 (64.8)	23 (35.9)	
*t* (15; 17)	11 (8.1)	3 (4.2)	8 (12.5)	
*t* (8; 21)	7 (5.2)	4 (5.6)	3 (4.7)	
*t* (9; 11)	1 (0.7)	1 (1.4)	0 (0.0)	
FAB classifications				0.048^∗^^,a^
M0	15 (10.0)	11 (14.5)	4 (5.4)	
M1	35 (23.3)	20 (26.3)	15 (20.3)	
M2	38 (25.3)	20 (26.3)	18 (24.3)	
M3	15 (10.0)	4 (5.3)	11 (14.9)	
M4	29 (19.3)	11 (14.5)	18 (24.3)	
M5	15 (10.0)	10 (13.2)	5 (6.8)	
M6	2 (1.3)	0 (0.0)	2 (2.7)	
M7	1 (0.7)	0 (0.0)	1 (1.4)	
FLT3 mutation				0.026^∗^
Negative	102 (69.4)	46 (60.5)	56 (78.9)	
Positive	45 (30.6)	30 (39.5)	15 (21.1)	
IDH1 R132 mutation				0.245^a^
Negative	136 (91.3)	66 (88.0)	70 (94.6)	
Positive	13 (8.7)	9 (12.0)	4 (5.4)	
IDH1 R140 mutation				0.369^a^
Negative	137 (91.9)	68 (89.5)	69 (94.5)	
Positive	12 (8.1)	8 (10.5)	4 (5.5)	
IDH1 R172 mutation				0.238^a^
Negative	147 (98.7)	76 (100.0)	71 (97.3)	
Positive	2 (1.3)	0 (0.0)	2 (2.7)	
RAS mutation				0.276^a^
Negative	142 (94.7)	70 (92.1)	72 (97.3)	
Positive	8 (5.3)	6 (7.9)	2 (2.7)	
NPM1 mutation				0.001^∗^
Negative	117 (78.0)	50 (65.8)	67 (90.5)	
Positive	33 (22.0)	26 (34.2)	7 (9.5)	
DNMT3A mutation				0.463
Negative	92 (84.4)	50 (87.7)	42 (80.8)	
Positive	17 (15.6)	7 (12.3)	10 (19.2)	
RUNX1 mutation				0.545^a^
Negative	97 (89.0)	52 (91.2)	45 (86.5)	
Positive	12 (11.0)	5 (8.8)	7 (13.5)	
WBC count (×10^9^/L)		31.00 [8.00,69.00]	11.50 [3.00,33.75]	0.001^∗^^,b^
BM blasts		49.00 [17.75,72.50]	17.00 [2.00,55.50]	<0.001^∗^^,b^
PB blasts		75.00 [61.50,86.25]	61.00 [40.00,81.00]	0.001^∗^^,b^

BM: bone marrow; PB: peripheral blood. ^∗^statistically significant, *P* < 0.05. ^a^Fisher's exact test. ^b^Wilcoxon rank sum test.

**Table 2:** Gene sets enriched in low-EPAS1 expression phenotype.

Gene set name	NES	ADJ *P* value	FDR
KEGG_RIBOSOME	-2.770	0.036	0.022
REACTOME_SRP_DEPENDENT_COTRANSLATIONAL_PROTEIN_TARGETING_TO_MEMBRANE	-2.661	0.045	0.028
ROSS_AML_WITH_MLL_FUSIONS	-2.564	0.034	0.021
VALK_AML_WITH_FLT3_ITD	-2.180	0.023	0.015
PARK_TRETINOIN_RESPONSE_AND_PML_RARA_FUSION	-2.113	0.021	0.013
REACTOME_HDACS_DEACETYLATE_HISTONES	-1.958	0.028	0.017

NES: normalized enrichment score; ADJ: adjusted; FDR: false discovery rate.

**Table 3:** Gene sets enriched in high-EPAS1 expression phenotype.

Gene set name	NES	ADJ *P* value	FDR
REACTOME_REGULATION_OF_ACTIN_DYNAMICS_FOR_PHAGOCYTIC_CUP_FORMATION	2.510	0.010	0.006
REACTOME_COMPLEMENT_CASCADE	2.427	0.010	0.006
REACTOME_SIGNALING_BY_THE_B_CELL_RECEPTOR_BCR	2.283	0.010	0.006
BOQUEST_STEM_CELL_UP	2.260	0.010	0.006
REACTOME_CELL_SURFACE_INTERACTIONS_AT_THE_VASCULAR_WALL	2.257	0.010	0.006
REACTOME_IMMUNOREGULATORY_INTERACTIONS_BETWEEN_A_LYMPHOID_AND_A_NON_LYMPHOID_CELL	2.174	0.010	0.006

NES: normalized enrichment score; ADJ: adjusted; FDR: false discovery rate.

**Table 4:** EPAS1 expression associated with clinicopathologic characteristics (logistic regression).

Characteristics	Total (*n*)	Odds ratio (OR)	*P* value
WBC count (×10^9/L) (>20 vs. <=20)	150	0.42 (0.22-0.80)	0.009^∗^
PB blasts (%) (>70 vs. <=70)	151	0.46 (0.24-0.88)	0.019^∗^
BM blasts (%) (>20 vs. <=20)	151	0.31 (0.16-0.61)	<0.001^∗^
Cytogenetic risk (poor vs. favorable & intermediate)	149	1.58 (0.75-3.43)	0.234
FLT3 mutation (positive vs. negative)	147	0.41 (0.19-0.84)	0.017^∗^
IDH1 R132 mutation (positive vs. negative)	149	0.42 (0.11-1.35)	0.164
IDH1 R140 mutation (positive vs. negative)	149	0.49 (0.13-1.64)	0.266
RAS mutation (positive vs. negative)	150	0.32 (0.05-1.46)	0.176
NPM1 mutation (positive vs. negative)	150	0.20 (0.08-0.48)	<0.001^∗^
DNMT3A mutation (positive vs. negative)	109	1.70 (0.60-5.05)	0.321
RUNX1 mutation (positive vs. negative)	109	1.62 (0.48-5.80)	0.438

PB: peripheral blood; BM: bone marrow. ^∗^statistically significant, *P* < 0.05.

**Table 5:** Univariate and multivariate Cox regression analyses of prognostic covariates in AML patients.

Characteristics	HR	95% CI	*P* value
Univariate analysis			
WBC count (×10^9/L) (>20 vs. <=20)	1.161	0.760-1.772	0.49
PB blasts (%) (>70 vs. <=70)	1.230	0.806-1.878	0.338
BM blasts (%) (>20 vs. <=20)	1.165	0.758-1.790	0.486
Cytogenetic risk (favorable vs. poor & intermediate)	0.312	0.160-0.606	<0.001
Gender (male vs. female)	1.030	0.674-1.572	0.892
Age (>60 vs. <=60)	3.333	2.164-5.134	<0.001
Race (White vs. Asian & Black or African American)	1.200	0.485-2.966	0.693
FLT3 mutation (positive vs. negative)	0.787	0.496-1.248	0.309
IDH1 R132 mutation (positive vs. negative)	1.702	0.689-4.205	0.249
IDH1 R140 mutation (positive vs. negative)	0.884	0.442-1.769	0.727
IDH1 R172 mutation (positive vs. negative)	1.641	0.228-11.804	0.623
RAS mutation (positive vs. negative)	1.555	0.568-4.254	0.39
NPM1 mutation (positive vs. negative)	0.879	0.546-1.416	0.596
DNMT3A mutation (positive vs. negative)	1.404	0.731-2.696	0.308
RUNX1 mutation (positive vs. negative)	1.119	0.553-2.267	0.754
EPAS1 (high vs. low)	0.542	0.352-0.836	0.006
Multivariate analysis			
Cytogenetic risk (favorable vs. poor & intermediate)	0.508	0.249-1.034	0.062
Age (>60 vs. <=60)	3.174	2.023-4.980	<0.001
EPAS1 (high vs. low)	0.577	0.364-0.915	0.019

HR: hazard ratio; CI: confidence intervals; PB: peripheral blood; BM: bone marrow.

## Data Availability

The data that support the findings of this study are available from the corresponding author upon request.

## Supplementary Materials

Supplementary 1 Supplementary Figure 1: differences in the enrichment scores of extracellular matrix-related pathways between high- and low-EPAS1 expression groups.

Supplementary 2 Supplementary Figure 2: top 25 genes differentially expressed in high- and low-EPAS1 expression groups and control group.

Supplementary 3 R codes used in our study.
