# Supplementary material for: Bioinformatics Analysis Identifies EPAS1 as a Novel Prognostic Marker Correlated with Immune Infiltration in Acute Myeloid Leukemia
Source: Dis Markers. 2023 Apr 17;2023:6072782. doi: 10.1155/2023/6072782 (PMC10137199; doi:10.1155/2023/6072782)
Supplement: Supplementary 1 — Supplementary Figure 1: differences in the enrichment scores of extracellular matrix-related pathways between high- and low-EPAS1 expression groups. [file 6072782.f1.pdf]

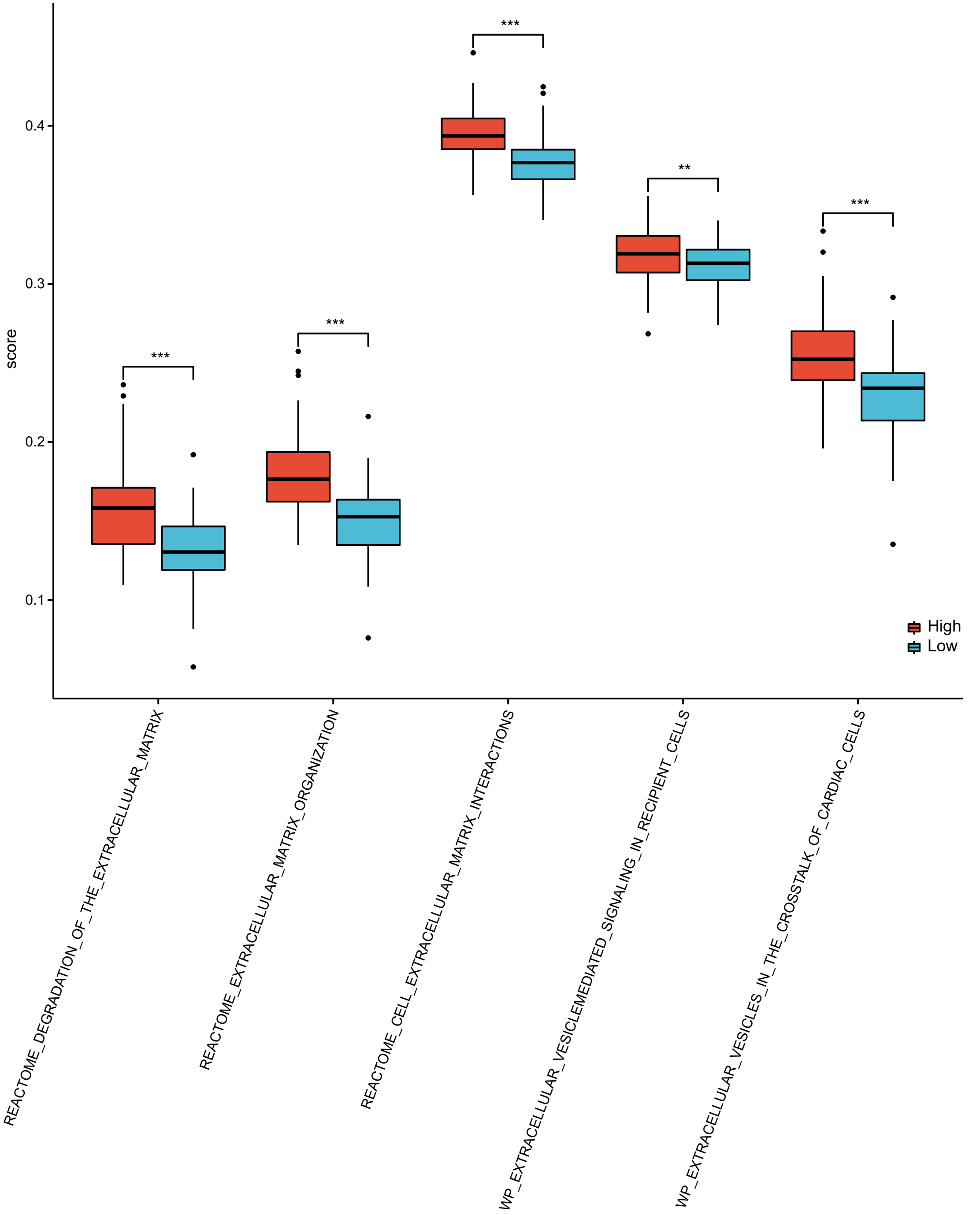

Supplementary Figure 1: Differences in the enrichment scores of extracellular matrix-related pathways between high and low EPAS1 expression groups.
