# Supplementary material for: Bioinformatics Analysis Identifies EPAS1 as a Novel Prognostic Marker Correlated with Immune Infiltration in Acute Myeloid Leukemia
Source: Dis Markers. 2023 Apr 17;2023:6072782. doi: 10.1155/2023/6072782 (PMC10137199; doi:10.1155/2023/6072782)
Supplement: Supplementary 2 — Supplementary Figure 2: top 25 genes differentially expressed in high- and low-EPAS1 expression groups and control group. [file 6072782.f2.pdf]

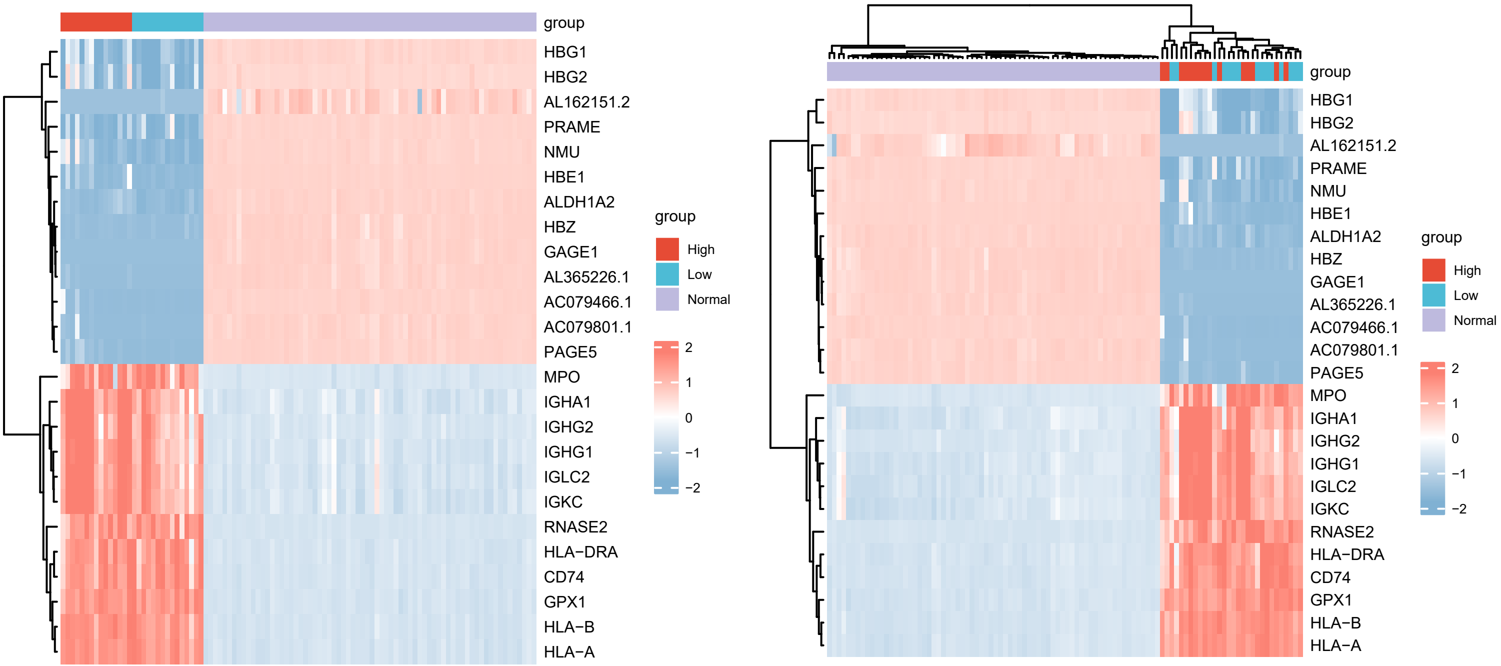

Supplementary Figure 2: Top 25 genes differentially expressed in high and low EPAS1 expression groups and normal control group.
